# An Information Theoretical Multilayer Network Approach to Breast Cancer Transcriptional Regulation

**DOI:** 10.3389/fgene.2021.617512

**Published:** 2021-03-18

**Authors:** Soledad Ochoa, Guillermo de Anda-Jáuregui, Enrique Hernández-Lemus

**Affiliations:** ^1^Computational Genomics Division, National Institute of Genomic Medicine, Mexico City, Mexico; ^2^Centro de Ciencias de la Complejidad, Universidad Nacional Autónoma de México, Mexico City, Mexico; ^3^Conacyt Research Chairs, National Council on Science and Technology, Mexico City, Mexico

**Keywords:** breast cancer, probabilistic multilayer networks, information theory, co-expression networks, multiomics analysis

## Abstract

Breast cancer is a complex, highly heterogeneous disease at multiple levels ranging from its genetic origins and molecular processes to clinical manifestations. This heterogeneity has given rise to the so-called intrinsic or molecular breast cancer subtypes. Aside from classification, these subtypes have set a basis for differential prognosis and treatment. Multiple regulatory mechanisms—involving a variety of biomolecular entities—suffer from alterations leading to the diseased phenotypes. Information theoretical approaches have been found to be useful in the description of these complex regulatory programs. In this work, we identified the interactions occurring between three main mechanisms of regulation of the gene expression program: transcription factor regulation, regulation via noncoding RNA, and epigenetic regulation through DNA methylation. Using data from The Cancer Genome Atlas, we inferred probabilistic multilayer networks, identifying key regulatory circuits able to (partially) explain the alterations that lead from a healthy phenotype to different manifestations of breast cancer, as captured by its molecular subtype classification. We also found some general trends in the topology of the multi-omic regulatory networks: Tumor subtype networks present longer shortest paths than their normal tissue counterpart; epigenomic regulation has frequently focused on genes enriched for certain biological processes; CpG methylation and miRNA interactions are often part of a regulatory core of conserved interactions. The use of probabilistic measures to infer information regarding theoretical-derived multilayer networks based on multi-omic high-throughput data is hence presented as a useful methodological approach to capture some of the molecular heterogeneity behind regulatory phenomena in breast cancer, and potentially other diseases.

## 1. Introduction

Cancer is a collection of complex diseases characterized by uncontrolled proliferation (GM., 2000). The complexity of cancer comes, among other sources, from the interaction of different molecular layers and the environment and results in both intra- and inter-tumor heterogeneity (Tian et al., 2011; Burrell et al., 2013; Turashvili and Brogi, 2017). In the case of breast cancer, this heterogeneity has been intended to be captured by tumor sub-classification. Breast cancer has been thus classified into subtypes with specific molecular signatures and treatment options (Prat et al., 2015), though each altered molecular layer groups differently (Cancer Genome Atlas Network, 2012). Some of these layers, such as gene expression and DNA methylation, have been intensively studied, while others like chromatin accessibility are still gaining attention (Liu, 2020). However, all these layers are interrelated (Wang et al., 2014) and the study of their collective effect calls for multi-omic approaches.

Multi-omic approaches have become possible only recently due to their more stringent methodological requirements. A (relatively large) minimal number of samples are required to find significant patterns, and the needed sample size increases with the noise added per each additional omic. Measurements must refer to the same set of samples, with sustained quality, no matter the differences in data type and range (Kristensen et al., 2014; Bersanelli et al., 2016; Tarazona et al., 2020).

The ability to model heterogeneous and high-dimensional data has made networks a promising tool for multi-omics integration (Vaske et al., 2010; Kim et al., 2012; Wang et al., 2014). For instance, mutual information (MI) networks combining miRNA and gene expressions have been built to gain insight on the regulatory mechanisms behind breast cancer (Drago-García et al., 2017). Such networks pinpointed miR-200 and miR-199 as regulators of the acquisition of epithelial and mesenchymal traits. Another example is the coupling of promoter methylation, transcription factors (TFs), and gene expression in several cancers proposed by Liu et al. Based on those networks, they fitted per target regression models that suggest key cancer processes are jointly regulated by TFs and CpG sites, not by either one alone. Those processes turned out to be different than the processes dominated by copy number variants (Liu et al., 2019).

Gene co-expression networks have been extensively studied in the context of breast cancer subtypes, both from our group (de Anda-Jáuregui et al., 2016; de Anda-Jáuregui et al., 2019; Espinal-Enriquez et al., 2017; Dorantes-Gilardi et al., 2020; García-Cortés et al., 2020; Ochoa et al., 2020) and others (Tang et al., 2018; Bhuva et al., 2019). Here, we are presenting the results on the incorporation of CpG methylation in addition to the study of coding transcripts (for both TFs and other genes) and miRNA expression analyzed in each breast cancer subtype. The goal is to identify CpG sites, TF transcripts (referred to as TF-genes from here on) and miRNAs associated with the biological processes differentially activated in breast cancer, since these may perform potential roles as regulators of the phenotype. Integrated analyses may thus provide us with additional hints toward the possible discovery of synergistic or cooperative effects of these different regulators.

## 2. Materials and Methods

### 2.1. Data Acquisition

Concurrent-sample measurements of DNA methylation, transcript abundance, and miRNA expression were downloaded from the GDC (https://portal.gdc.cancer.gov/repository) in May 2019. Samples quantified with the Illumina Human Methylation 27 BeadChip, which covers a smaller portion of the genome, were discarded. Instead, we used data obtained with the Infinium HumanMethylation450 BeadChip, which covers 99% of RefSeq genes, at both transcription repressive sites around promoters and transcription favorable sites on the body of genes (Dedeurwaerder et al., 2011). Since these measurements pertain to three distinct techniques: methylation beadchip, RNAseq, and miRNAseq; we treat them as separate omics, here on identified as CpG sites, transcripts, and miRNAs. By including the whole set of features, we wanted to recover the highest possible number of interactions. Subtype classification was also downloaded from the GDC metadata using the TCGABiolinks R package (Colaprico et al., 2016).

Each omic was pre-processed independently according to Aryee et al. (2014), Tarazona et al. (2015), and Tam et al. (2015) by using biomaRt v95. Preprocessing included filtering of transcripts and miRNAs with low counts, TMM normalization and batch effect correction with ARSyN. Low count thresholds are less than 10 counts per million for transcripts and, less than 5 counts for 25% or more of the samples for every subtype, in the case of miRNAs. Transcripts were also normalized for length and GC content via full method. Annotation was downloaded to tag transcripts coding for TFs (TF-genes).

For methylation data, we discarded sites with over 75% missing values, nonmapped or located within sexual chromosomes or SNPs. Remaining missing values were imputed via nearest neighbors. Resulting beta value matrices were transformed into *M*-value matrices. This way, values of 384,575 methylation probes, 16,475 coding transcripts, and 433 miRNA precursors were obtained for 45 unique samples belonging to the Her2+ subtype, 395 of LumA, 128 of LumB, and 125 of Basal subtypes, plus 75 samples of nontumor (normal adjacent) tissue. All samples correspond to women, ranging in age at diagnosis between 26 and 91 years, and further details can be found in the Supplementary File 1.

### 2.2. Inference of MI Networks

Normalized data matrices for methylation data, coding transcripts, and miRNA expression were merged by sample and used as input to the MI-based ARACNE network deconvolution algorithm (Margolin et al., 2006).

ARACNE calculates mutual information between every pair of features and returns values above a threshold, set either as an MI value or as a permutation *p*-value. There is no restriction on the features that get paired by MI calculation, and it was not required for CpG sites to be on the same chromosome than targets, nor that target promoters carry some TF motif. The only restriction made was for CpG-CpG interactions, which were not calculated due to the space needed to save all possible combinatoria. In a nutshell, pairwise mutual information calculations were performed for the expression patterns for all genes and miRNAs, as well as the beta values for genomewide CpG methylation. Co-expression networks on the different layers were built from the most significant interactions as follows:

Since MI distribution has been shown to change depending on the type of molecules (Drago-García et al., 2017), a unique threshold cannot be set. A unique MI threshold has the risk of discarding significant interactions between molecules whose values simply fall in a lower range or accepting nonsignificant interaction between molecules exhibiting values on a higher than the threshold range. A threshold based on *p*-values induces a similar problem because MI and *p*-values are roughly inversely proportional. For example, it is possible to see that setting the threshold value to 0.1 in **Figure 2C** would discard most miRNA to transcript interactions while retaining all the interactions among transcripts, and that such pruning of edges would affect differently the distinct networks, producing disparate results due to methodology. Mutual information distributions and their respective threshold values have a direct impact on the topology of the underlying networks and in particular in the degree distributions. So, by choosing MI cutoffs one is indeed imposing an associated network topology.

To overcome this issue, top 10,000 interaction for each type of molecules paired were selected, that is, the 10,000 interactions with the highest MI values linking CpG sites and transcripts (both genes and TF-genes), CpG sites and miRNAs, transcripts (both genes and TF-genes) and miRNAs, and interactions within these two last groups. This way, the topology resulting from such a set of interactions is comparable among cancer subtypes and normal tissue. Thus, we take the focus from the varying MI distributions to a defined topology size. This strategy has been previously validated and used by our group for the reconstruction of biologically relevant networks from high-throughput data (de Anda-Jáuregui et al., 2016).

Fixed bandwidth ARACNE calculations ran with kernel width parameter (h) of 0.165024 for Basal data, 0.211612 for Her2+, 0.12527 for LumA, 0.16567 for LumB, and 0.18679 for normal tissue. To check the significance of the interactions in these networks, maximal MI for each pair of molecules was registered for different *p*-value thresholds. Thresholds with MI values larger than those observed in a network contain the network's interactions. The *p*-value upper limits for the final networks are reported in Supplementary Table 1. Finally, MI distributions were compared via Kolmogorov-Smirnov tests with False Discovery Rate (FDR) correction.

Kernel width variation between subtypes can be attributed to the size of the datasets. We estimated z-scores with subsets of the data to evaluate how size differences are affecting the networks. To this end, 100 subsamples of size 45 were taken from luminal and Basal subtypes, and from the normal tissue data. The subsample size was set to 45 for direct comparison with Her2-associated networks. MI was calculated using these subsets and resulting distributions served for z-score calculation. Results can be observed in Supplementary Table 2.

By keeping the same number of links in each layer, we are able to directly compare network parameters between layers. However, it should be noted that since the number of possible links increases (quadratically) with the number of nodes, there may be differences in the statistical significance. However, all our networks have an equivalent *p*-value of less than 1E-6 (corresponding to the CpG layer in Her2+ samples, i.e., the layer with more features analyzed for the subtype with the lowest number of samples).

### 2.3. Functional Enrichment

Independently of network construction, differential expression vs. normal tissue was calculated for every subtype using limma's treat (McCarthy and Smyth, 2009) function with null fold change equal to 1.5. Afterwards, the complete rank of differential expression *t*-values was used as input for a GSEA on each subtype, as implemented in the R package fgsea (Sergushichev, 2016), vs. the biological process gene ontology.

Processes with Benjamini and Hochberg adjusted *p*-value lesser than 0.01 were subject to over-representation analysis on the corresponding subtype network. Processes with Benjamini and Hochberg adjusted *p*-value over 0.05 were regarded as nonrepresented in the network. The rest was examined for CpG sites, miRNAs, and TF-genes associated via their MI value with the functionally annotated transcripts, since these serve as potential regulators of the function. For the normal tissue, all the processes significant for a subtype were submitted to the over-representation analysis. There are processes present in a subtype network, but absent from the normal tissue network. This results in a total of 176 processes over-represented in at least one subtype network, from which only 128 have a match in the normal tissue network. In this step, a mean of 59.05% nodes was removed from the MI networks, a breakdown of which can be found in Supplementary Table 3.

Resulting networks were visualized using Cytoscape (Shannon et al., 2003) with a prefuse force directed layout. Nodes were added to account for the enriched functions in order to find out which biological processes were potentially regulated. Hereafter, these networks are denominated as *final networks* or *functionally enriched networks* to distinguish them from the purely probabilistically inferred networks. These focus on the processes whose expression is the most associated with the subtype, and that rely on interactions with the highest MI; these functions are potentially relevant for the subtypes and so it may be useful to elucidate the associated regulatory patterns.

### 2.4. Validation of MI Interactions

To check for additional support for the interactions in the final networks, regulator-target databases were reviewed per omic. CpG annotation was taken from Illumina's manifest file, and the genes affected by each site are considered as *validated*. CpG sites on the same chromosome than the target gene are considered as plausible regulators and regarded when adding predictions. These are distinguished from one another as *mapped* and *same chromosome* sites in Supplementary Table 1.

Transcription factor targets were downloaded via tftargets https://github.com/slowkow/tftargets, a package that queries TRED, ITFP, ENCODE, and TRRUST databases, and the lists compiled by (Neph et al., 2012; Marbach et al., 2016). Only TRRUST TF-targets are considered as validated, since those were manually curated from PubMed articles. The associations between transcripts and miRNAs were sought on DIANA-microT-CDS, ElMMo, MicroCosm, miRanda, miRDB, PicTar, PITA, TargetScan, miRecords, miRTarBase, and TarBase via multiMiR (Ru et al., 2014).

Targets for both TF and miRNA were searched in the tables obtained from each package. The only tuning needed for TF's search was to track ENTREZ gene IDs, HGNC symbols, and Ensembl IDs; this was done according to biomaRt data. Since GDC measurements are identified by precursor miRNA IDs, while databases use mature miRNA tags, this search requires translation from one to the other using mirBase records.

### 2.5. Characterization of the Potential Regulators

Looking for differences between subtypes, total regulators of each type were added for every process. Retrieved counts were compared between each subtype and the normal tissue via Fisher tests with FDR correction. Enrichment is only considered if the process has associated regulators of any type, in both the normal tissue and the subtype under evaluation. Statistical tests were one-tailed. Null hypothesis is set to be opposite to expected trends, that is, “greater” for the CpG nodes and “less” for both TF-genes and miRNAs.

To weight the abundance of each regulatory layer, counts per regulator type were divided by the total number of regulators associated with the process, obtaining the percentages displayed in Supplementary File 2.

Node topological parameters were calculated over the MI networks, that is, ignoring the *biological processes* nodes, which have to be excluded given the different nature of their associated edges: probabilistically inferred or database curated. Distributions were compared via Wilcoxon rank sum test with continuity correction and *p*-values were FDR corrected.

### 2.6. Potential Regulators Comparison

Both intra and inter-subtype comparisons were made. To this end, Jaccard index was calculated for each pair of processes from the same subtype for the intra-subtype comparison and for the same process in different subtypes for the inter-subtype comparison. Inter-subtype contrasts count edges instead of nodes, because in this case, the interest is on conserved regulatory interactions. Obtained distributions were evaluated via Kolmogorov–Smirnov tests with FDR correction.

The number of potential regulators either shared or exclusive between processes of the same subtype was evaluated via Fisher tests with the corresponding alternative hypothesis set “greater” for the CpG sites, and “less” for TF-genes and miRNAs, as previously stated.

All the code used for the described analysis is available at https://github.com/CSB-IG/MI-MultiOmics.git.

## 3. Results

MI networks were constructed for each breast cancer subtype and for normal tissue combining three different omics: CpG methylation, transcript gene expression, and miRNA expression. The second omic includes two layers of information, regulated genes, and TF-genes. No restriction was made on the features that can get paired by MI calculation, CpG sites can get linked to targets on a different chromosome, and TFs may associate with targets without the akin binding motifs. Let us recall that mutual information does not assume any a priori mechanism and relies instead on statistical dependencies. Table 1 presents all the different networks of MI-inferred potential gene regulators (CpG-mRNA, TF-gene-mRNA, miRNA-mRNA, mRNA-mRNA) plus the biological processes associated with them.

**Table 1:** Networks description.

**Edges**	**Basal**	**Her2+**	**LumA**	**LumB**	**Normal**
CpG–mRNA	2,456	3,847	1,932	4,334	4,732
	(554)	(88)	(536)	(708)	(28)
TF-genes–mRNA	2,735	2,498	1,686	2,746	2,544
	(5)	(2)	(5)	(1)	(14)
miRNA–mRNA	3,483	3,889	2,065	4,074	4,953
	(167)	(226)	(111)	(201)	(284)
mRNA–mRNA	4,189	4,523	2,276	4,709	5,088
**Nodes**					
Biological processes	109	119	34	123	128
CpG sites	2,254	3,769	1,553	3,638	3,863
Transcripts	4,567	6,356	2,834	5,235	4,733
TF-genes	658	748	375	618	684
miRNAs	433	432	408	433	14

*Validated interactions appear between parentheses. Edges correspond to significant statistical dependencies inferred via MI calculations*.

MI networks went through two pruning steps, first by edge significance (see section 2.2) and then by functional annotation of the nodes (see section 2.3). The first one retains only the most significant interactions, i.e., those with the largest MI. For the second pruning, biological processes with significant GSEA enrichment scores were mapped to the networks, keeping only the nodes involved in an enriched process and their first neighbors. For the normal tissue, all the processes significant for a subtype were subjected to over-representation analysis. This way, only nodes linked to transcripts involved in a process altered in the subtype are kept. Then, final networks carry only CpG–transcript, miRNA–transcript, and transcript–transcript interactions with the highest MI. The hypothesis is that nodes with gene expression regulatory roles may regulate the associated biological process. This would be partially explained, if regulators co-vary (even in a nonlinear fashion) with their targets, thus becoming detectable as MI statistical dependencies. It is relevant to recall, however, that regulatory mechanisms are proxied here by the information given by the omics under study. Other regulatory mechanisms—including those of (explicit) chromatin remodeling, as well as post-transcriptional and post-translational modifications among others—may not be fully accounted by the statistical dependencies structures just outlined.

To assess the contribution of linear correlation measures, we are including further calculations in Supplementary Figures 1–5 to show how many of the MI edges would be lost if the criterion was instead an FDR-corrected Pearson correlation with an associated *p* < 0.05.

**Figure 1:**
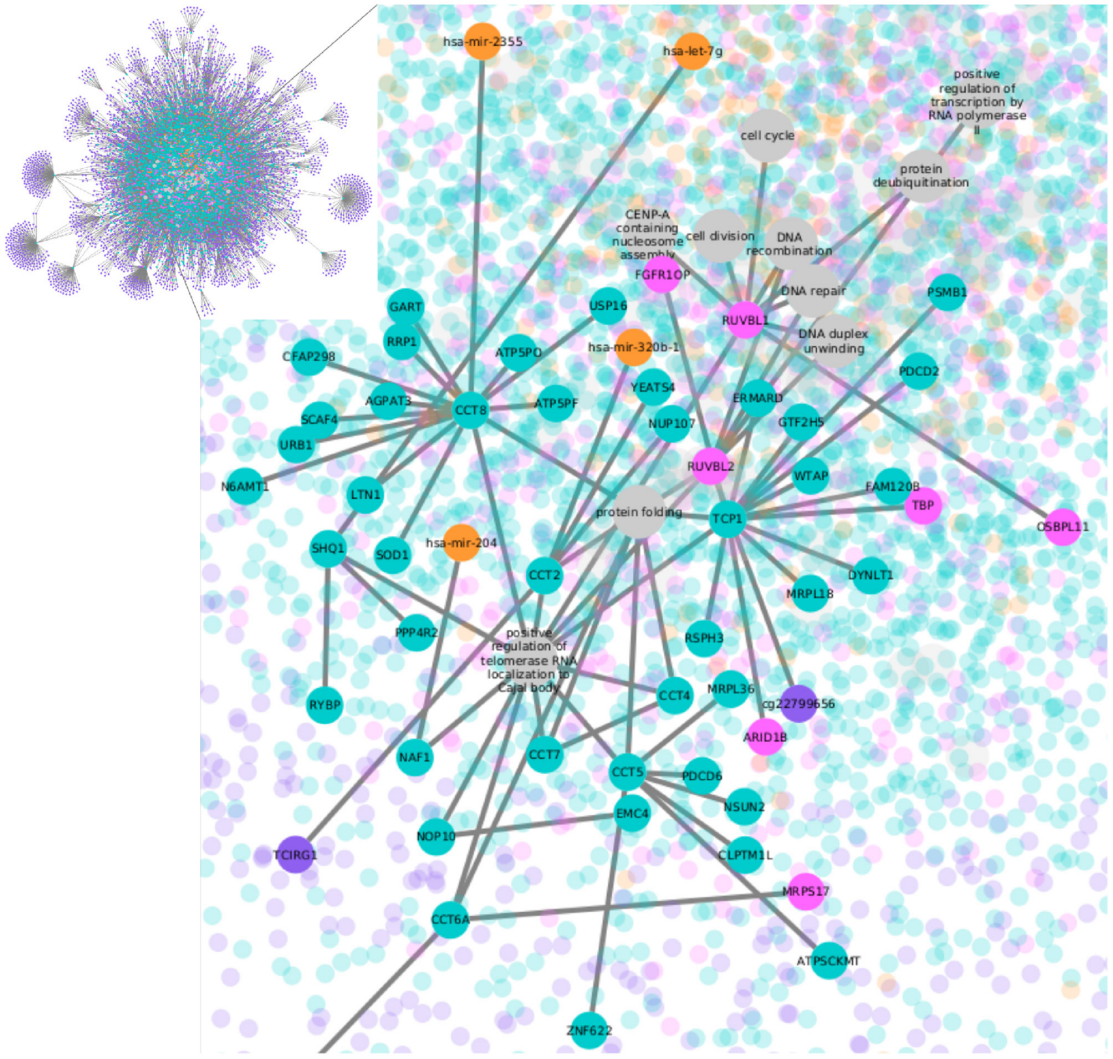
LumB subtype network. Nodes represent CpG sites in purple, transcripts in green, TF-genes in pink, miRNAs in orange, and biological processes in gray. The whole network is shown in the upper left box; the rest of the figure contains a zoom-in.

To identify unequivocally the functions linked to each transcript, nodes representing the biological processes were added, resulting in multipartite graphs as the one shown in Figure 1. The multipartite nature of the network comes from the three different molecules (CpG sites, transcripts, and miRNAs) associated with the biological process nodes. There are also two kinds of edges: (1) MI edges, which indicate molecule covariation, and (2) functional annotation edges, which make explicit the link of a transcript and a process. All the five networks, four for the breast cancer subtypes and one for the normal tissue, consist of one giant single connected component. As expected, CpG methylation, which has the largest number of features, is the most represented omic in the networks.

By contrasting the molecules paired with databases on regulator-target, we can see how many of the found interactions were already known. Interactions absent from the databases can be new, previously unknown relationships, or simply indirect associations caused by the statistical co-variation of the molecules. Between 1.67 and 11.47% of the interactions linking a transcript with a potential regulator, that is a CpG, a TF-gene, or a miRNA, have been validated. The number of validated edges per subtype is shown in Table 1. If predictions are included (see section 2.4), 8.26–23.52% of the interactions have additional support. The effect on the networks of considering only some of the potential regulatory CpGs can be seen in Supplementary Figure 6. A large number of TF target predictions are based on ChIP-seq experiments, not necessarily performed on breast tissue, which may lower such matches.

Having described the general features of the five networks (one for each tumor subtype plus the one for normal tissue), we proceeded to search for differences between the behavior of the different omics among subtypes. Focus was made on differences on the potential regulators, since this could translate to regulatory features behind the subtypes.

### 3.1. Network Parameters Vary Between Omics

As stated earlier, there are two types of edges in the networks, edges that account for co-expression (i.e., significant statistical dependency) with a given value of MI, and edges that record functional annotation as presented in curated databases. Given the difference of meaning, interactions need to be analyzed separately.

**Figure 2:**
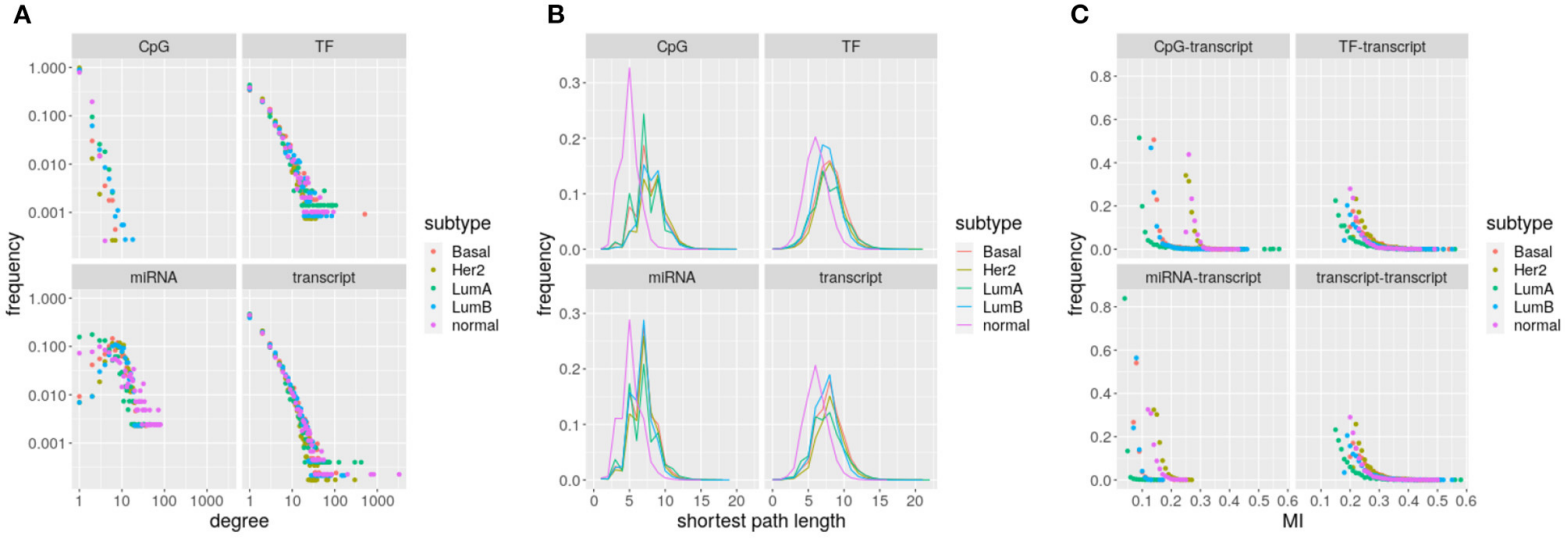
Networks parameters: **(A)** degree, **(B)** shortest path, and **(C)** edges with mutual information. The subtype depicted by each network is indicated by the color code.

Focusing only on MI edges, the number of components grows from 1 to hundreds. Average degree is around 3 for all the networks, but distributions vary between omics (Wilcoxon rank sum test *q*-value ≤ 1.666712e-22, Figure 2A). Though TF-genes and gene transcripts are measured by the same omic, distributions are significantly different (Wilcoxon rank sum test *q*-value ≤ 0.0237) for the five networks. The case of miRNAs stands out because distributions are not scale-free like. CpG sites show the lowest degrees, with an average of 89.42% nodes connected only with another node. Thus, most CpG sites do not contribute to network communication as they do not interlink paths.

The constrained (bounded) degree distribution of CpGs translates into a large portion of unreachable target nodes, an average of 32.23% of targets cannot be reached from some CpGs. Consistently, miRNAs have an average of 19.71% of unreachable targets, which is the lowest frequency. Despite range similarity, distributions change significantly across omics and between tumor subtypes and normal tissue (Wilcoxon rank sum test *q*-value ≃ 0). Again, distributions for TF-genes and gene transcripts are significantly different (see Figure 2B, Wilcoxon rank sum test *q*-value ≤ 0.0002). The shift in the position of the peak in breast cancer subtypes relative to normal tissue suggests a loss of communication.

Edges also differ depending on the omics involved. Differences on mutual information distributions between omics and subtypes are significant (Kolmogorov–Smirnov *q*-value ≤ 5.53264e-06). TF-genes and gene transcripts follow the same distribution on each network. It is noticeable how small is the range of miRNA interactions and how CpG distributions segregate.

In Table 1 and Figure 2, we have characterized the interactions occurring within and between different omics in each molecular subtype of breast cancer. We may appreciate that both intra-layer and inter-layer interaction sets are specific to each biological condition. In what follows, we will now leverage both the monolayer and multilayer interactions to further elucidate biological functions associated with each molecular subtype.

### 3.2. Representation of Potential Regulators Changes With the Subtype

**Figure 3:**
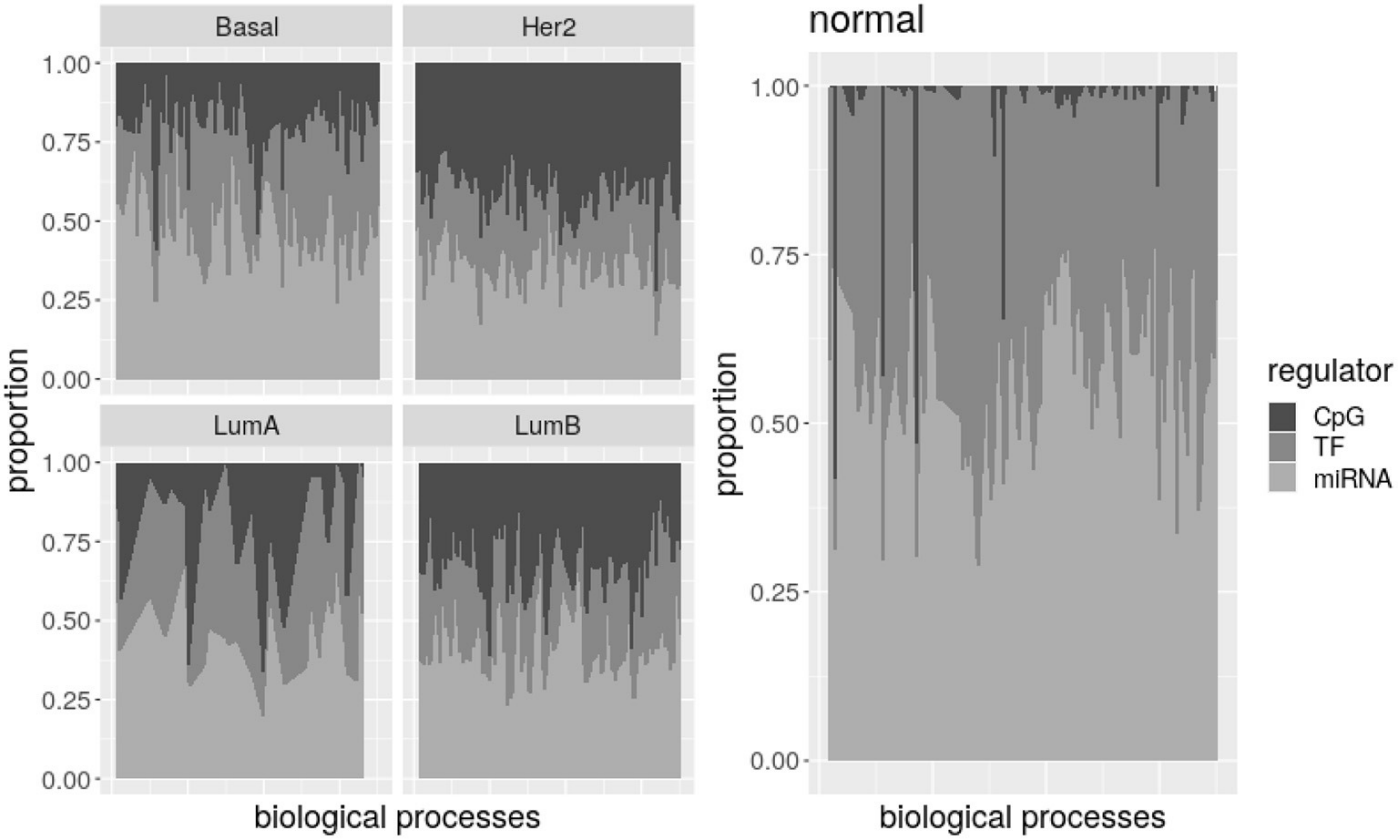
Potential regulators per biological process. There is an area plot per network. Each column is a process. The proportion of regulators of each type associated with the process is denoted by the grayscale. All the processes together show how common are the three potential regulators in the subtype.

To further explore the differences among potential regulators, its abundance per biological processes was calculated. To this end, total number of CpG, TF-genes, and miRNA nodes were obtained for each biological process. The proportion of regulators of each type is shown in Figure 3 as a simple measure of the impact a regulatory layer has in a given subtype. A version of this figure with labels for biological processes and the corresponding table are available as Supplementary Material.

Despite variability, it is evident that the number of CpG nodes increases on breast cancer subtypes relative to normal tissue, while TF-genes and miRNA numbers of nodes are lower. The plot for Luminal A subtype is less noisy because this subtype has less processes on its network. Nevertheless, by comparing processes represented in each subtype and normal tissue, we found most processes are significantly enriched of CpG nodes in the Basal, Her2+, and LumB subtypes. Simultaneously, TF-genes and miRNAs are significantly under-represented on more than half of the processes in the Her2+ and LumB networks. Additionally, between 20 and 33% of the Basal- and LumA-associated processes show under-representation of TF-genes and miRNAs, and almost half of LumA processes are enriched of CpG nodes.

If potential regulators are actually regulating their associated processes, this may indicate transcriptional and post-transcriptional regulations are subdue in breast cancer subtypes while epigenetic regulation gains strength. By considering the combined effect across layers (inter-layer regulation) as well as the effects on a single type of molecular interaction, as given by each omic dataset (intra-layer regulation), it is possible to develop a deeper understanding of cross-regulatory effects. This will be considered in the next subsections in the context of the different tumor subtypes.

### 3.3. Normal Interactions With Potential Regulators Are Almost Absent in Breast Cancer Networks

Having seen that the abundance of complete regulatory layers is not maintained across subtypes, we wondered what happens to specific regulatory interactions. With this in mind, we calculated the extent to which interactions with potential regulators are shared among networks by calculating their associated Jaccard indices. The Jaccard index weights the size of the intersection between two sets with the size of their union. In other words, it counts what fraction of the elements is shared from the total. This way, sets of different extensions are assigned values between 0 and 1, and can be objectively compared.

**Figure 4:**
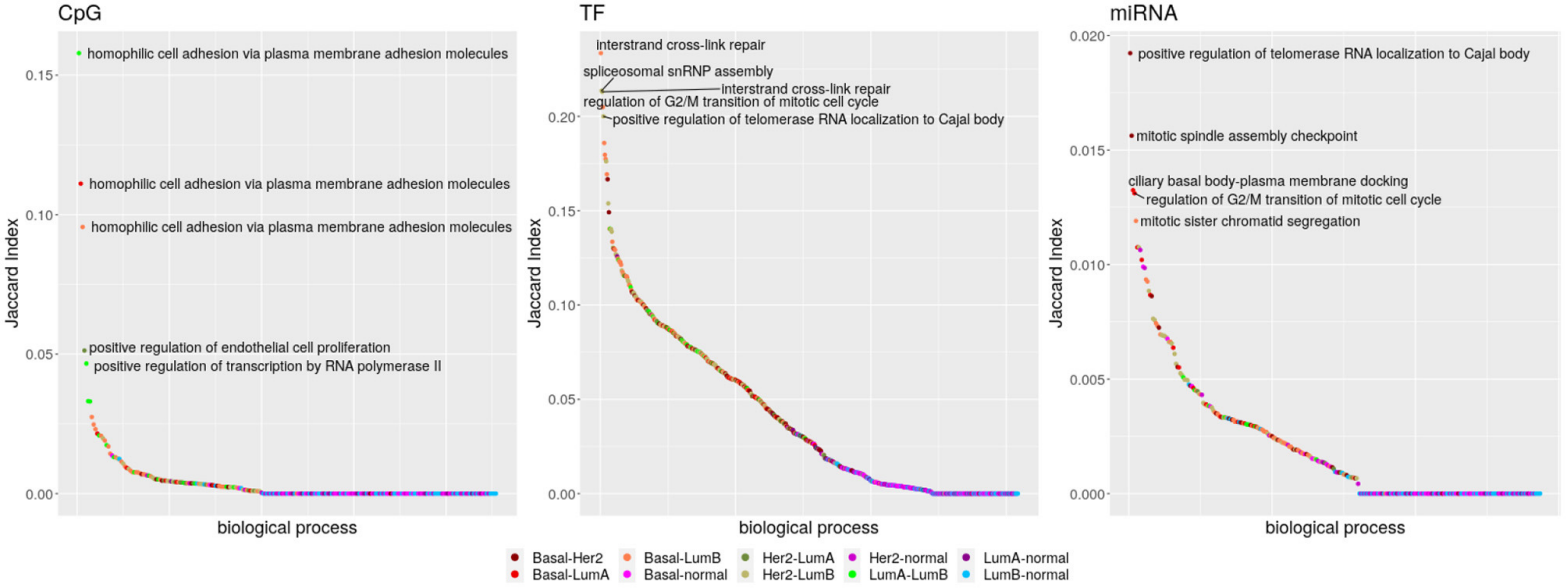
Inter-subtype sharing. Biological processes are symbolized by dots, ordered from those sharing more potentially regulatory interactions to those sharing less. The color code indicates which networks are contrasted. Comparisons with the Basal network are in reddish colors, and those with Her2+ are in dry greens; indexes involving the normal tissue go from pink to blue and those between the luminal subtypes are in bright green.

From the total of 176 biological processes enriched in any network, 86.36% appear in at least two subtypes and also are able to share edges. Interactions with miRNAs are poorly shared, while TF-genes and CpG-edges reach a similar maximum but following different distributions (Kolmogorov–Smirnov test *q*-value ≤ 2.498002e-16). Links with any regulator are almost not shared between the breast cancer subtypes and the normal tissue (Kolmogorov–Smirnov test *q*-value ≤ 1.541449e-06), but TF-genes are visibly more shared. The five biological processes with the highest Jaccard index are shown in Figure 4.

**Figure 5:**
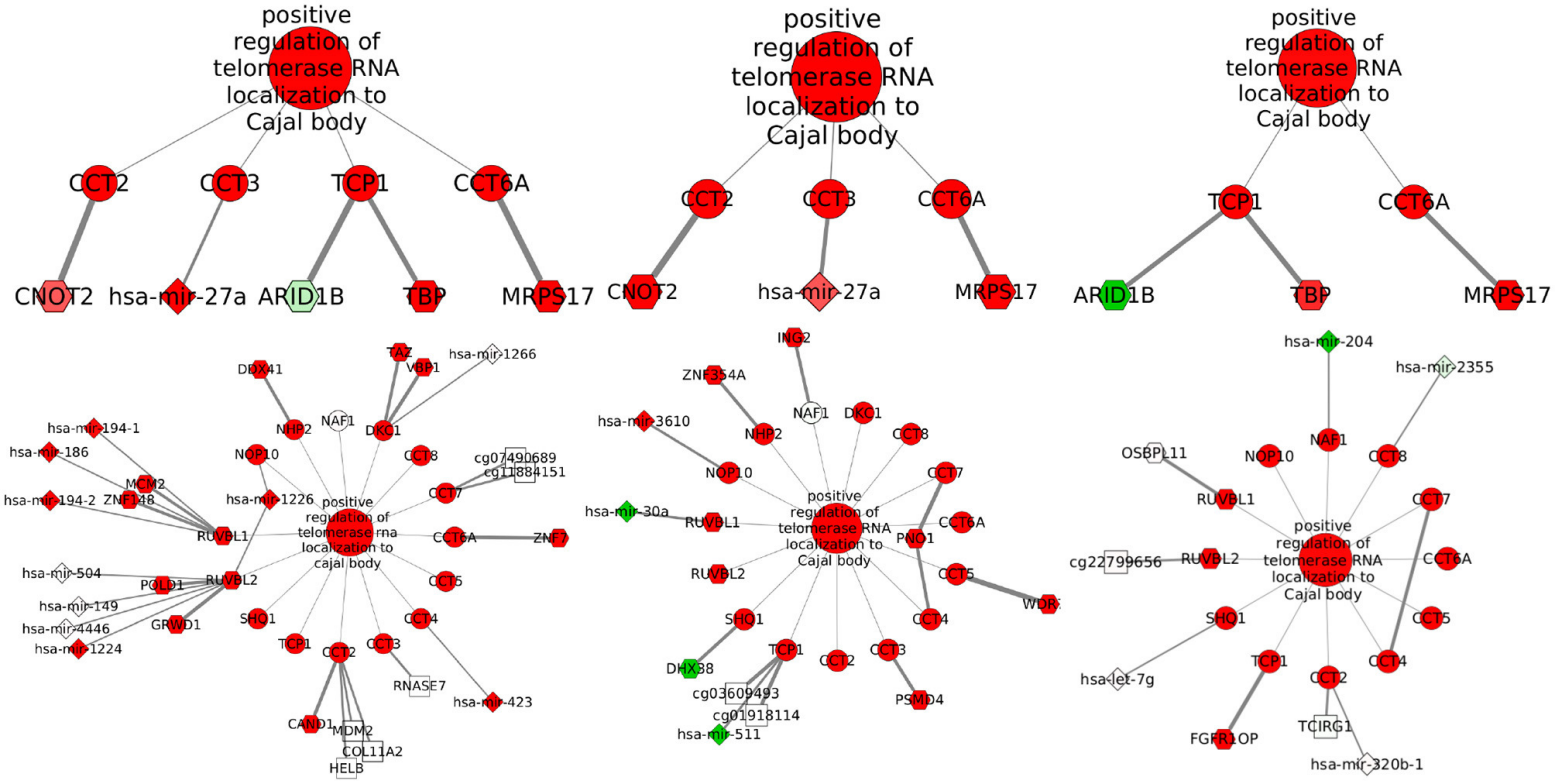
Potential regulators associated with telomerase localization to Cajal body. From left to right, subgraphs with shared **(top)** and exclusive **(bottom)** interactions are shown for Basal, Her2+, and LumB subtypes. Nodes are colored in red if differential expression values or GSEA normalized enrichment scores are positive or in green if values are negative. Node transparency represents statistical significance. Nodes for biological processes and transcripts are circles, TF-genes are hexagons, miRNAs are diamonds, and CpG sites are squares. When the altered gene is known, its name is on the CpG label. Edge thickness represents MI values.

Localization of telomerase RNA (hTR) to the Cajal body has the highest index for miRNAs for the sharing among Basal and Her2+ networks. This process is also the fifth for TF-genes, but pairing Her2+ and LumB. Figure 5 shows that the elevated Jaccard indices are driven by only few shared interactions among sets of small size. Although potential regulation changes, the process is equivalently activated in these three subtypes. The interaction linking Chaperonin Containing TCP1 Subunit 6A (CCT6) with Mitochondrial Ribosomal Protein S17 (MRPS17) is shared across these three subtypes, but may be an artifact of the physical proximity of the genes.

### 3.4. Within Subtypes, CpG Nodes Are Exclusive of Processes, but miRNAs Do Not

For a complementary perspective, we checked if regulators are shared between the distinct biological processes enriched in a single subtype. Degree distributions suggest that CpG sites are exclusive, while miRNAs and TF-genes are shared.

**Figure 6:**
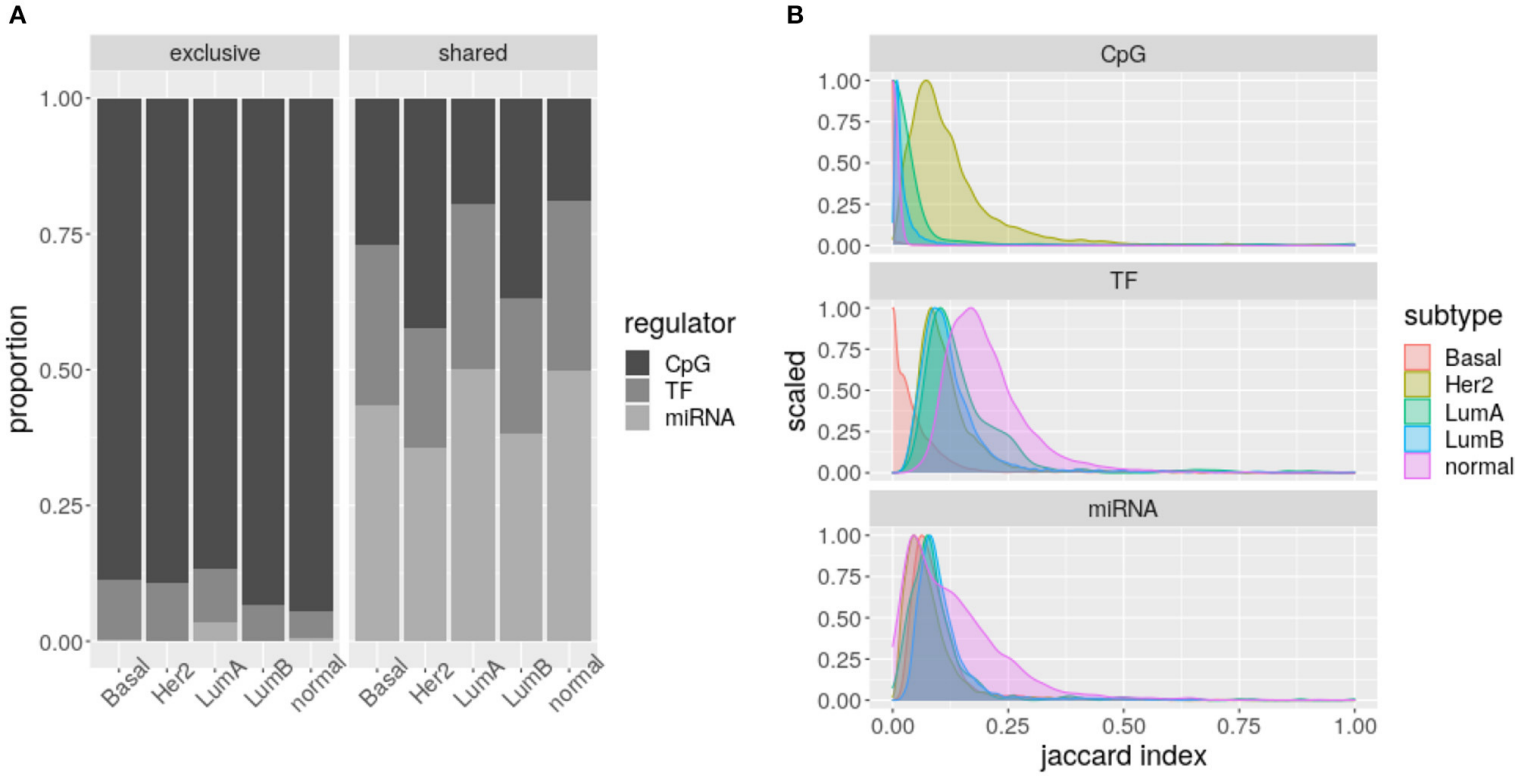
Intra-subtype sharing: **(A)** Proportion of regulators shared or exclusive, across biological processes per subtype. Each column represents a different network. The grayscale indicates the potential regulator. **(B)** Jaccard Index distributions per regulator. The color code indicates the network represented.

Figure 6A shows how CpG sites are mostly exclusive of one biological process (Fisher test *q*-value ≤ 1.949349e-67), while TF-genes and specially miRNAs are shared between various processes (Fisher test *q*-value ≤ 1.411310e-11). That is, miRNA expression seems to connect different biological processes, while for CpG methylation this effect is much lower.

When calculating the Jaccard index of the biological processes enriched for each subtype and regulator, significantly different distributions are obtained (Kolmogorov–Smirnov test *q*-value ≤ 0.0221, Figure 6B). Consistently, as presented in Figure 6A, these distributions show CpG sites are less shared, but TF-genes seem to be more shared than miRNAs. The CpG sites of Her2+ and the TF-genes of Basal subtypes call for attention.

## 4. Discussion

With the aim of exploring potential regulatory patterns of breast cancer subtype expression, we reconstructed via mutual information, multi-omics networks, functionally enriched in GO biological processes. The hypothesis is that there may be a transitive property between the regulators of a transcript and the function associated with the transcript.

This way, potential regulators emerging from the networks are associated with the biological processes significantly enriched. Potential regulators separate domains topologically from non-regulatory transcripts and from each other. Degree distributions are coherent with the pattern of exclusivity and sharing across processes, observed later for CpG sites and TF-genes–miRNAs, respectively. Both results coincide with what is known for the molecule types. Namely, CpG sites have a rather local effect (Li and Zhang, 2014), while TF-genes and miRNAs are *promiscuous*, spanning through a much wider chromosome range (Cho, 2007).

Given the pattern of sharing/exclusivity across processes, one could expect that targeting DNA methylation may drive focused changes, while miRNAs and TF-genes targeting may show pleiotropy. However, current modulators of DNA methylation act over the whole genome, making impossible to change sites related to specific processes. On the contrary, CpG sites linked to specific processes may have potential as predictors of process alteration. Such potential is promising given the early timing of methylation alterations in other cancer types (Vrba and Futscher, 2019). For example, there are 19 CpG sites associated with DNA damage checkpoint in Her2+ subtype, suggesting a possible monitoring mechanism. Nevertheless, it would be necessary to have a whole new project to test the predictability of such sites. The value of the multilayer networks presented here is to propose this kind of hypothesis among all possible combinations, though they need further testing.

To verify that CpG exclusivity per process is not induced by the omission of CpG–miRNA and miRNA–miRNA interactions, non-functionally enriched networks were revisited (Supplementary Figure 7). Distributions still change per omic (Wilcoxon rank sum test *q*-value ≤ 4.657478e-16), while the percentage of CpG nodes with degree equal to one is maintained above 90%, indicating that observations made for the first neighbors are relevant when considering farther neighbors. By considering the top 10,000 MI interactions per paired molecules, we observed that CpG sites do not significantly participate in the regulatory circuitry flow but are often endpoints.

Shortest-paths distributions point out to a decrease in communication independently of the omic observed. This is in line with the under-representation of TF-genes and miRNAs detected specially in Her2+ and Luminal B associated processes. To reconcile communication reduction with over-representation of CpG sites on the subtypes, it is necessary to remember that most CpG nodes do not participate in network connection. These layer level patterns consistently match literature reports on alteration of CpG methylation (Cancer Genome Atlas Network, 2012; Berger et al., 2018), and miRNA expression in breast cancer (O'Day and Lal, 2010; Bertoli et al., 2015; Klinge, 2018).

Two subtype-specific patterns attracted our attention, elevated sharing of CpG nodes between the processes enriched for the subtype Her2+, and decreased sharing of Basal TF-genes. The 2,112 CpG sites shared by Her2+ processes are all over the genome, with a slight increase in chromosomes 1 and 17. While chromosome 1 has been reported as severely affected by differential methylation (Lindqvist et al., 2014), the characteristic amplification of chromosome 17 cannot be fully accounted for the excess sharing. Only 76 from the 1576 genes affected by shared CpG sites co-amplify with the *Her2* gene. Similarly, only 22.91% of affected genes have evidence of AR regulation, a TF postulated to crosstalk with Her2 amplification (Daemen and Manning, 2018).

The other pattern that caught our attention is the decrease in TF-genes linking any two processes in the network for the Basal subtype. This is not caused by a decrease in TF-genes, since the quantity of TF-gene nodes associated with the processes is equivalent for all the networks. Uniqueness of biological processes in the Basal network are neither responsible, seeing that only 6 processes are exclusive for this subtype. Instead, we speculate the pattern is related to promoter accessibility because of ATAC-seq data groups tumors in Basal and non-basal networks (Corces et al., 2018). Further characterization finds a pro-metastasis open-chromatin signature elevated in the Basal subtype (Cai et al., 2020). By its side, protein level measures integrated with copy number normalized gene expression suggest TF-genes as relevant drivers of this subtype (Koh et al., 2019).

Only one edge level pattern was found, but it is a remarkable one. Interactions with regulatory potential are poorly shared among all networks, but the edges of the normal tissue network are almost endemic, especially in the case of CpG sites and miRNAs. If we conform to the idea that DNA methylation preserves cell type identity (Szyf, 2012), our results advert mammary gland defining methylation has been lost in processes like T-cell receptor signaling pathway and inflammatory response.

Localization of hTR to the Cajal body is a biological process linked with cancer cell's unlimited division, given that these organelles have been implicated in the biogenesis of telomerase (Tomlinson et al., 2008). Associated subgraphs exhibit how few edges are shared across subtypes and suggest a convergence of different regulatory schemes to a single outcome. The relative uniformity of enrichment scores across subtypes (Supplementary Figure 8) indicates this could be common. Such pattern is important because the way a tumor gains an expression signature might create different vulnerabilities. An example is given by tumors compatible with Her2-enriched expression, but lacking the mutation that makes tumors sensitive to targeted treatment (Godoy-Ortiz et al., 2019).

We must, however, stress that one limitation of the current approach resides on the relatively small sample size. This is a constraint due to lack of availability of a larger dataset comprising the same types of multi-omic data. Limited availability of additional independent datasets also precluded us to validate our findings on an independent cohort. To partially alleviate this, we have resorted to subsampling procedures and null models. The effect of data size differences can be seen in Supplementary Figure 9 and Supplementary Table 2. Supplementary Table 2 and Supplementary Figure 9 show the dispersion between MI values estimated with the whole set of samples as well as values obtained through subsampling, for the interactions with the lowest, most varying significance, those between miRNAs and transcripts. Though subsampling repetition is low (100), it catches a tendency toward small z-scores and noisier low subsampled MI values. This means higher z-scores are not necessarily bad, since the large difference between complete and subsampled values maintains points at the top of the range. Altogether, subsampling suggests adding samples would reach higher MI values, but would not alter the ranking dramatically, which supports the (cautious) usage of datasets such as the one used for Her2+. Nevertheless, our analysis could only take advantage of an increase of the number available samples.

As with other areas of molecular biology, one driving force behind the development of multi-omics is the expectation that the results from these technologies may lead to novel pharmacological interventions (de Anda-Jáuregui and Hernández-Lemus, 2020). Nevertheless, the translation from the identification of a perturbation to clinical implementation is not straightforward (Silverman et al., 2020). In this regard, pharmaceutical interventions in each of the analyzed layers are unevenly distributed: drugs that have effects on epigenetic modifications such as methylation have not attained the efficacy that was expected (Buocikova et al., 2020), although they remain an important research area. Meanwhile, gene expression has been able to identify biomarkers as well as drug repurposing opportunities (Mejía-Pedroza et al., 2018; Koudijs et al., 2019). In this context, the type of analyses that we present here provides the opportunity to identify not only the deregulation features in each regulatory layer but also the way it connects to other molecular elements. As such, the opportunity to modulate virtually undruggable targets through the control of its neighbors may help unblock therapeutic opportunities. However, as we mentioned previously, the path from these initial data analyses toward a translational and eventually a clinical setting is long and not necessarily direct.

### 4.1. Summary of Findings

In brief, the main findings that have been derived from our analysis may be summarized as follows:

- For networks associated with tumor subtypes:
  - Shortest paths are longer for the four subtypes than for the normal tissue.
  - Most biological processes (over 85%) are enriched for CpG nodes in Basal, Her2+, and LumB. Only 41.38% of the processes in LumA are enriched for CpG nodes.
  - Most biological processes (over 50%) are under-represented of TF-gene and miRNA nodes in Her2+ and LumB.
  - Interactions with CpGs and miRNAs found in normal tissue network are near endemic.
  - Her2+ CpG nodes are more shared between processes than expected.
  - Basal TF-gene nodes are less shared between processes than expected.
- For differences in the representation of different omics:
  - CpG nodes tend to show degree = 1, which translates into exclusivity for each process.
  - TF-genes have fewer nodes with degree = 1, and miRNAs have even less. Consistently, these nodes are more shared between processes thus participating in concerted network communication.
  - miRNAs degree distribution shape is remarkably different.
- For shared interactions:
  - Those with CpGs and miRNAs are less maintained than those with TF-genes.

## 5. Conclusions

Together, the observations made from multi-omic mutual information networks for the different breast cancer subtypes build a landscape of the differential influence the distinct regulatory layers may exert over the phenotypes. This expands our understanding of breast cancer associated regulatory phenomena and poses possible treatment alternatives to be further explored. For example, now that there is evidence that CpG methylation coordinates with the expression of Her2-associated genes involved in most biological processes more than in any other subtype, experiments with de-methylation agents on this specific subtype seem relevant to analyze.

So far, the interaction between regulatory layers has been overlooked due to the paucity of data and inadequacy of methods. Yet, mutual information calculations and the available algorithms just presented have no formal restriction to handle different omics, unlike other correlation measures MI allows to handle variables with disparate dynamic ranges as it relies in the probability distributions, and has proven capable to retrieve single omics regulatory interactions. Results obtained with the multi-omic setting are encouraging, though refinement of post-MI analysis is needed and is indeed a further avenue of research within our group.

In order to capture CpG methylation and miRNAs linked to biological processes via the interaction with one another, a more sophisticated method would be needed. For example, a computationally expensive recovery of all the paths between transcripts associated with functions. Another possible improvement would be the implementation of a multi-omics data processing inequality (DPI). DPI states that the edge with the smaller MI in a triangle can be filtered out as indirect. However, MI distribution changes for every type of omics paired complicating MI comparisons. Perhaps a better alternative will be to resort to tensor representations of probabilistic multilayer networks (Hernández-Lemus, 2020).

It is also pertinent to recall that higher mutual information does not translate into causal interactions. The so-called *potential regulators* may simply co-vary with transcript expression, or causality may be dependent on an intermediate node. Even if linked CpGs sites regulated gene expression, omics that are not included like copy number variation may also play relevant roles. To identify the potential regulators whose patterns are most related to transcripts expression, there are other strategies available (Lê Cao et al., 2009), which may benefit from MI interaction scores (Koh et al., 2019). There are however more insights to be extracted from the multi-omics networks yet.

With the set of potential regulators associated with a biological process, we aspire to multi-layer regulatory models that include examples like the one described for miRNA processing enzymes Drosha and Dicer (Rupaimoole et al., 2014). Here, we present general results, but particular cases can be further examined within this general approach. When the focus is on particular models, the distinct regulators connected to single gene allow the proposal of hypothesis about synergy and antagonism among regulation layers. Nevertheless, this approach calls for a much more detailed scrutiny.

All in all, due to the relative simplicity and generalizability of the approach, the use of combined probabilistic modeling and knowledge discovery in databases presented here allows for the inference of regulatory models that may be refined by resorting to more specialized techniques, both experimental and computational.

## Data Availability Statement

The original contributions presented in the study are included in the article/Supplementary Material, further inquiries can be directed to the corresponding author/s.

## Author Contributions

SO organized data, developed code, performed calculations, analyzed data, and drafted the manuscript. GA-J contributed to the methodological approach, analyzed data, discussed results, and co-supervised the project. EH-L envisioned the project, devised the methodological strategy, designed the study, contributed to the methodological approach, analyzed data, discussed results, reviewed the manuscript, and supervised the project. All authors read and approved the final manuscript.

## Conflict of Interest

The authors declare that the research was conducted in the absence of any commercial or financial relationships that could be construed as a potential conflict of interest.
