# Supplementary material for: An Information Theoretical Multilayer Network Approach to Breast Cancer Transcriptional Regulation
Source: Front Genet. 2021 Mar 18;12:617512. doi: 10.3389/fgene.2021.617512 (PMC8014033; doi:10.3389/fgene.2021.617512)
Supplement: Supplementary file 3 [file Data_Sheet_1.PDF]

Table 1: Description of the edges in each network

edges	Basal	Her2	LumA	LumB	normal
CpG-mRNA	2456	3847	1932	4334	4732
mapped	554	88	536	708	28
same chromosome	1269	435	938	2145	235
TF-mRNA	2735	2498	1686	2746	2544
validated	5	2	5	1	14
predicted	127	121	135	133	282
miRNA-mRNA	3483	3889	2065	4074	4953
predicted	112	160	65	143	195
validated	167	226	111	201	284
p-values					
CpG-mRNA	$\leq 10^{-6}$	$\leq 10^{-6}$	$\leq 10^{-6}$	$\leq 10^{-6}$	$\leq 10^{-6}$
mRNA-mRNA	$\leq 10^{-6}$	$\leq 10^{-6}$	$\leq 10^{-6}$	$\leq 10^{-6}$	$\leq 10^{-6}$
miRNA-mRNA	$\leq 10^{-3}$	$\leq 10^{-4}$	$\leq 10^{-4}$	$\leq 10^{-3}$	$\leq 10^{-4}$

Table 2: Z-scores contrasting MI values obtained with the whole datasets and with subsamples

	Basal	LumA	LumB	normal
Min.	-0.7065	-0.9516	-0.555	-0.4561
1st Qu.	-0.1155	-0.5702	-0.1191	0.0478
Median	0.0249	-0.4872	0.0195	0.1793
Mean	0.0439	-0.4754	0.0376	0.2161
3rd Qu.	0.1873	-0.3936	0.1765	0.3509
Max.	1.3787	0.9084	1.1368	1.3527

Table 3: Classification of the nodes filtered at functional enrichment step.

	Basal	Her2	LumA	LumB	normal
CpG	10241	10997	10712	9319	4084
transcript	4355	4749	5388	4419	1947
miRNA	0	1	25	0	13

Near 10000 MI edges are not compliant with significance threshold (FDR corrected Pearson correlation with associated p-value below 0.05) for the subtypes, with Her2 networks being the most affected, and the networks from normal tissue missing only 3639 edges. These edges do not distribute uniformly among categories, while transcript to transcript edges are barely affected, up to 62.32% of miRNA-transcript MI edges for the LumA subtype are non significant for Pearson correlation. This comes as no surprise since MI p values vary too with the type of edge. Edges between transcripts, or involving CpG sites have p-values under 10^{-6}, but miRNA-transcript edges reach p-values of

10^{-3}. Then, what happens when the inference method is substituted, depends on the type of edge. CpG sites and transcripts are not greatly affected, and with them, functional annotation. On the other hand miRNAs could suffer from a change in the method. This is however, somehow expected since our current understanding of miRNA interactions point out to the existence of non-linear dependencies in the expression levels.

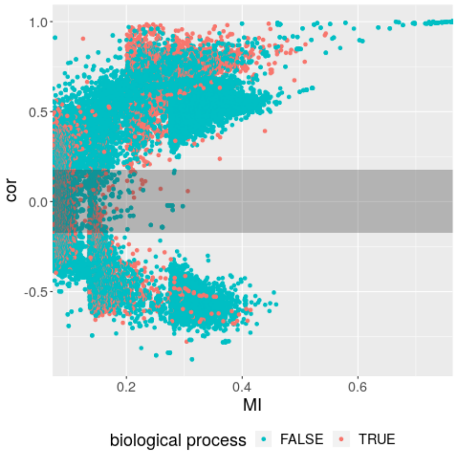

Figure 1: Pearson correlation of the edges in Basal networks. Points within the gray area have non-significant (≥ 0.05) correlation p-value

Table 4: Classification of the edges non-significant for Pearson correlation. Cells contain percentages per network and edge type

	top	final
mRNA-CpG	4.6262	13.4772
mRNA-mRNA	0.1951	0.1589
miRNA-CpG	0.0100	-
miRNA-mRNA	61.3166	60.7809
miRNA-miRNA	24.6000	-

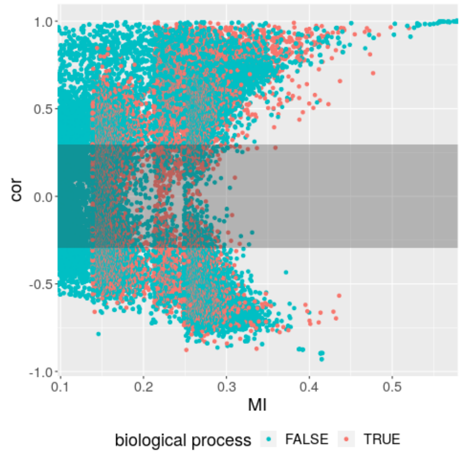

Figure 2: Pearson correlation of the edges in Her2 networks. Points within the gray area have non-significant ($\geq$ 0.05) correlation p-value

Table 5: Classification of the edges non-significant for Pearson correlation. Cells contain percentages per network and edge type

	top	final
mRNA-CpG	8.0106	5.4311
mRNA-mRNA	8.6571	4.3108
miRNA-CpG	0.53	-
miRNA-mRNA	54.8445	33.5150
miRNA-miRNA	44.96	-

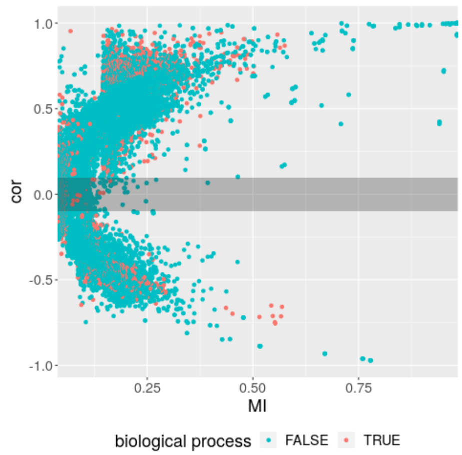

Figure 3: Pearson correlation of the edges in LumA networks. Points within the gray area have non-significant ($\geq$ 0.05) correlation p-value

Table 6: Classification of the edges non-significant for Pearson correlation. Cells contain percentages per network and edge type

	top	final
mRNA-CpG	15.3198	36.2836
mRNA-mRNA	0.3478	0.0252
miRNA-CpG	0	-
miRNA-mRNA	62.3188	60.339
miRNA-miRNA	11.24	-

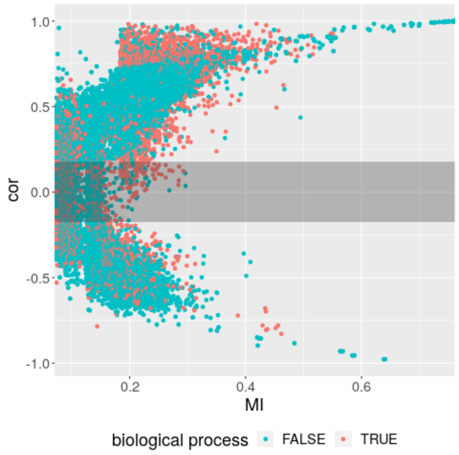

Figure 4: Pearson correlation of the edges in LumB networks. Points within the gray area have non-significant (≥ 0.05) correlation p-value

Table 7: Classification of the edges non-significant for Pearson correlation. Cells contain percentages per network and edge type

	top	final
mRNA-CpG	17.5256	17.3973
mRNA-mRNA	1.2181	1.3548
miRNA-CpG	0.05	-
miRNA-mRNA	57.0874	55.1055
miRNA-miRNA	22.7	-

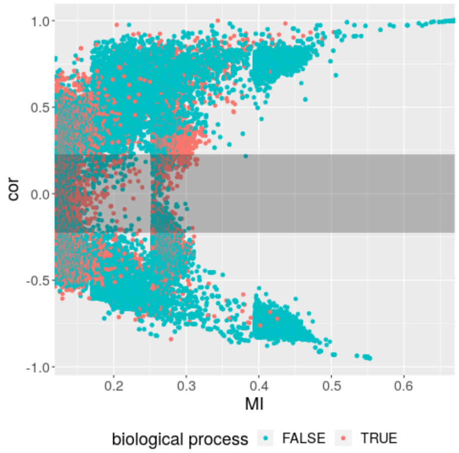

Figure 5: Pearson correlation of the edges in normal tissue networks. Points within the gray area have non-significant (≥ 0.05) correlation p-value

Table 8: Classification of the edges non-significant for Pearson correlation. Cells contain percentages per network and edge type

	top	final
mRNA-CpG	11.1997	7.9036
mRNA-mRNA	0.5068	0.3669
miRNA-CpG	0	-
miRNA-mRNA	23.4199	22.9154
miRNA-miRNA	1.86	-

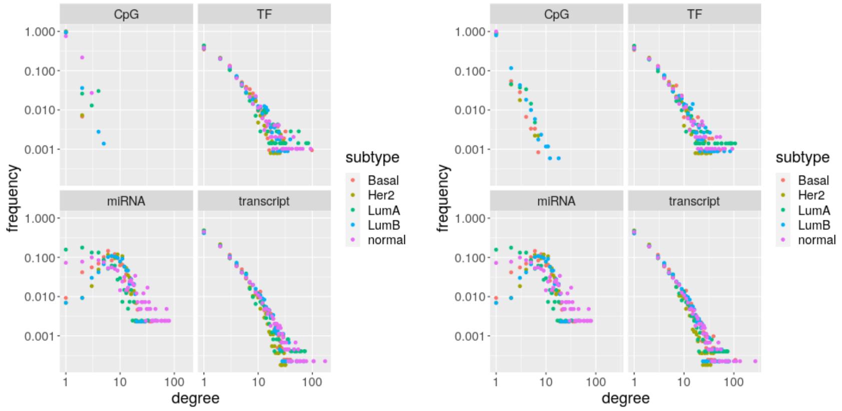

(a) (b)

Figure 6: Difference on degree when CpGs are constrained to (a) be differentially methylated or, (b) to be on the same chromosome than target genes.

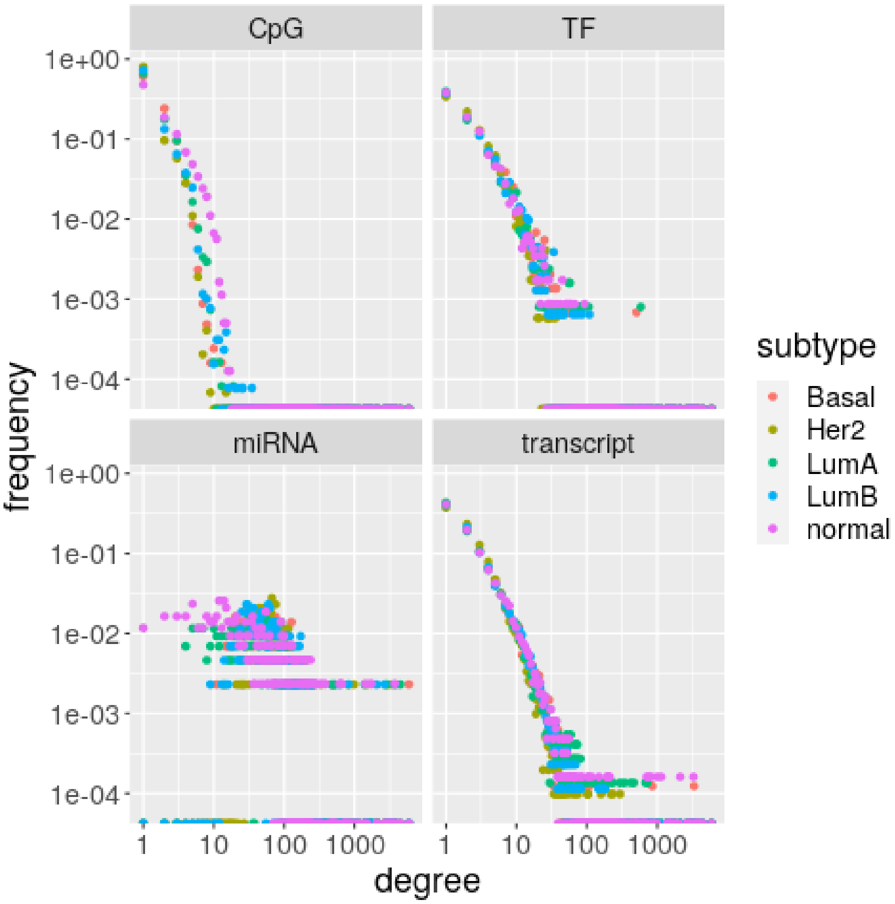

Figure 7: Degree distributions of the pre-functional enrichment networks

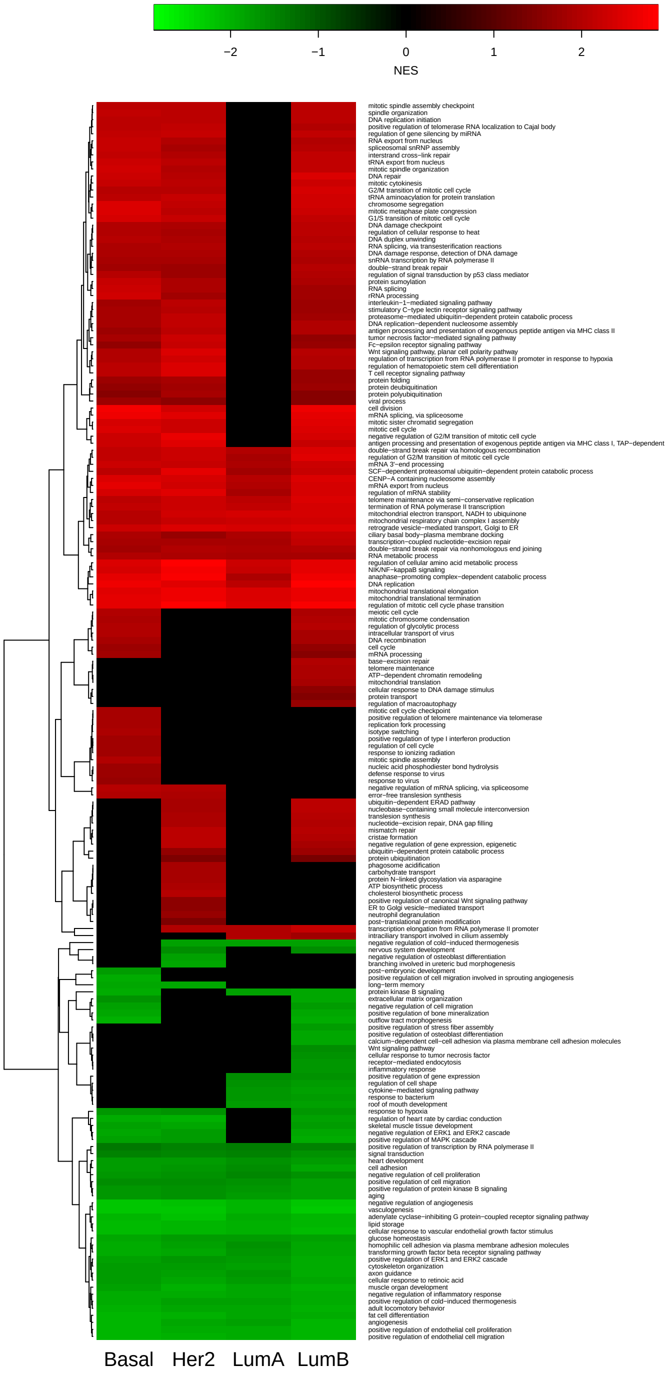

Figure 8: GSEA scores per process for the four subtypes

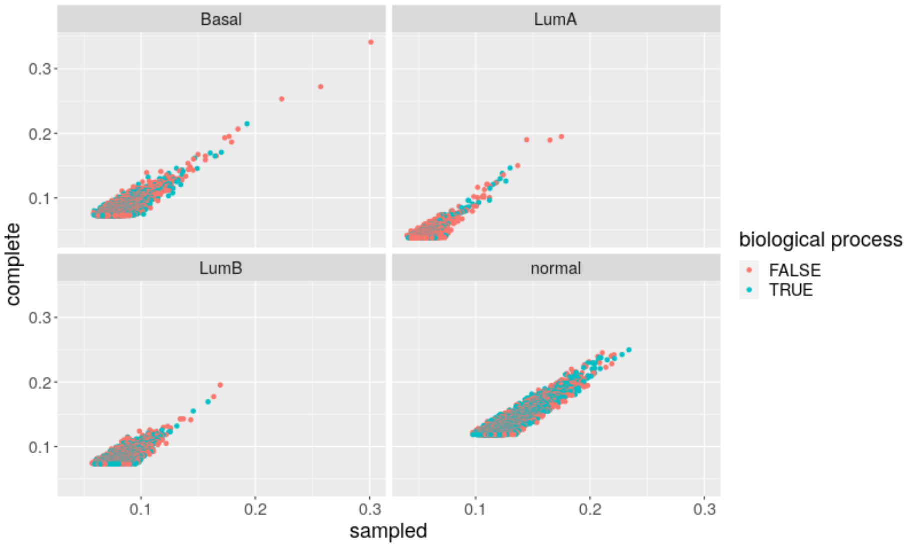

Figure 9: MI calculated with the complete data set against the mean subsampled value
