# Supplementary figures and images for: An Information Theoretical Multilayer Network Approach to Breast Cancer Transcriptional Regulation
Source: Front Genet. 2021 Mar 18;12:617512. doi: 10.3389/fgene.2021.617512 (PMC8014033; doi:10.3389/fgene.2021.617512)

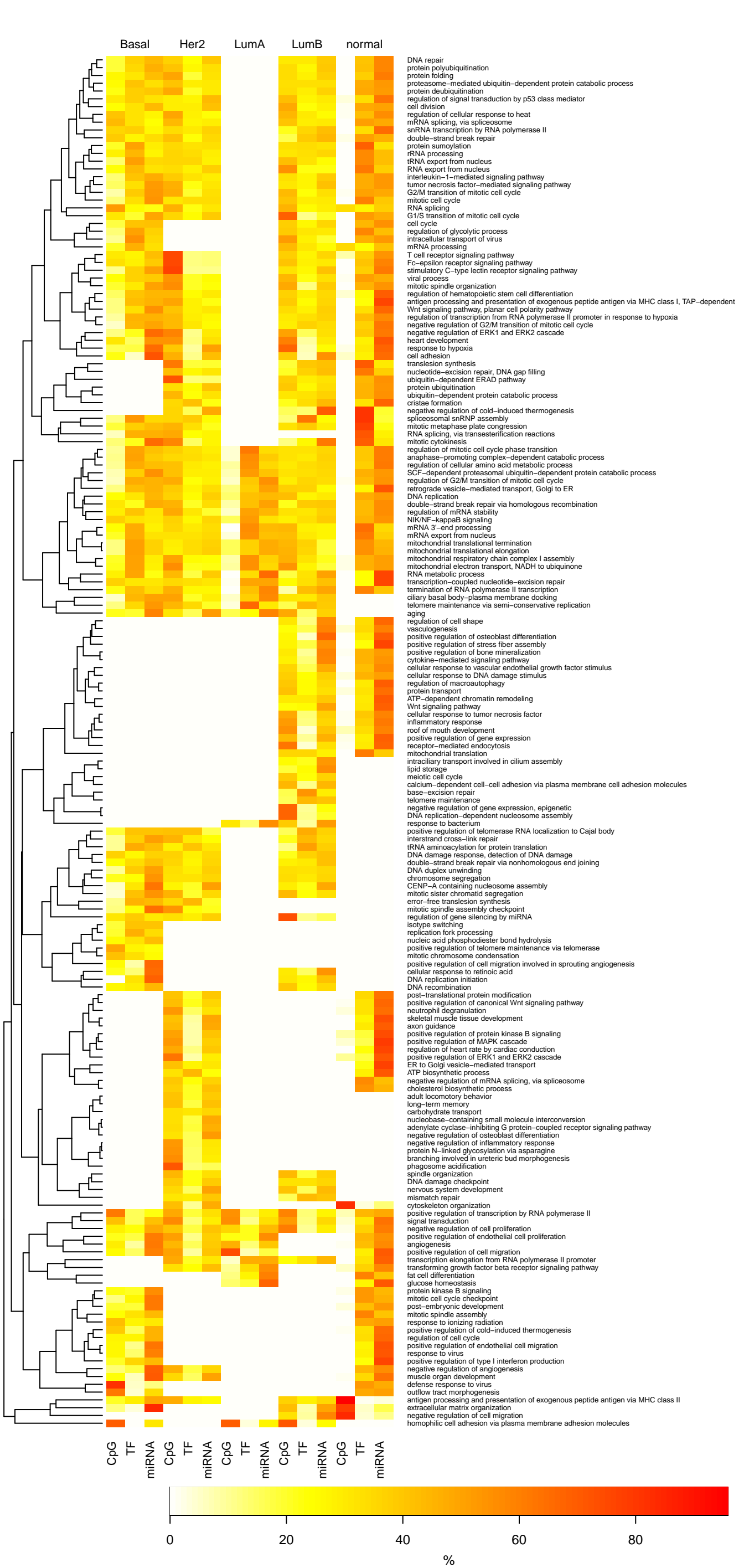

Supplement: Supplementary file 4 [file Data_Sheet_2.PDF]
